# Rapamycin Nano-Micelle Ophthalmic Solution Reduces Corneal Allograft Rejection by Potentiating Myeloid-Derived Suppressor Cells' Function

**DOI:** 10.3389/fimmu.2018.02283

**Published:** 2018-10-08

**Authors:** Chao Wei, Yuexin Wang, Li Ma, Xin Wang, Hao Chi, Sai Zhang, Ting Liu, Zhiyuan Li, Demeng Xiang, Yanling Dong, Xianggen Wu, Weiyun Shi, Hua Gao

**Affiliations:** ^1^State Key Laboratory Cultivation Base, Shandong Provincial Key Laboratory of Ophthalmology, Shandong Eye Institute, Shandong Academy of Medical Sciences, Qingdao, China; ^2^School of Medicine and Life Sciences, University of Jinan-Shandong Academy of Medical Sciences, Jinan, China; ^3^Qingdao University Medical College, Qingdao, China; ^4^Department of Pharmacy, College of Chemical Engineering, Qingdao University of Science and Technology, Qingdao, China

**Keywords:** rapamycin, corneal allograft rejection, myeloid-derived suppressor cells, immunosuppression, iNOS, Arg-1

## Abstract

Allograft rejection is the major cause of corneal allograft failure. Rapamycin (RAPA) has been reported as an effective and novel immunosuppressive agent for patients undergoing corneal transplantation. However, its high water insolubility and low bioavailability have strongly constrained its clinical application. In this study, we successfully developed a RAPA nano-micelle ophthalmic solution and found that corneal allograft survival in recipients treated with RAPA nano-micelle ophthalmic solution was significantly prolonged for more than 2 months, with less inflammatory infiltration, decreased production of pro-inflammatory factors, and elevated recruitment of myeloid-derived suppressor cells (MDSCs). MDSCs from mice treated with RAPA nano-micelle ophthalmic solution could significantly inhibit the proliferation of CD4^+^T cells through increased expressions of inducible nitric oxidase (iNOS) and arginase-1 (Arg-1). The activity blockade of Arg-1 and iNOS pharmacologically reversed their immunosuppressive ability. Moreover, the effects of RAPA were antagonized by the administration of anti-Gr-1 antibody or by inhibiting the activity of iNOS pharmacologically. In addition, RAPA nano-micelle also effectively alleviated allograft rejection in high-risk rabbit penetrating keratoplasty (PKP) models with corneal vascularization. Collectively, our results demonstrate that RAPA nano-micelle ophthalmic solution could improve the immunosuppressive activity of MDSCs through elevated expression of Arg-1 and iNOS, which highlights the possible therapeutic applications of RAPA against corneal allograft rejection.

## Introduction

Corneal diseases are the second leading cause of blindness globally (1). Corneal transplantation is the most common surgical procedure for restoring visual function. Although the immune privilege of corneal allografts endows a higher success rate in corneal transplantation than in other solid-organ transplantation, graft rejection remains the leading cause of human corneal allograft failure (2, 3). In clinical practice, immunosuppressive drugs, such as corticosteroids, cyclosporine A (CsA), and FK506 are widely topically applied to control the recipient's immune system and reduce the risk of corneal rejection (4, 5). However, the probability of penetrating corneal-graft survival in the whole cohort of studies was 87, 73, 60, and 46% at 1, 5, 10, and 15 years, respectively (6). Moreover, the success rate in high-risk patients with corneal neovascularization drops dramatically to as low as 20–40% (7, 8). Thus, the currently available immunosuppressants are still unsatisfactory for anti-rejection management after corneal transplantation.

Rapamycin (RAPA), a macrolide antibiotic initially discovered in 1975, has potent anti-proliferative, anti-inflammatory, anti-aging, and anti-tumor properties (9–11). Accumulative evidence demonstrates that RAPA also has strong immunosuppressive properties, and it is extensively used as an anti-rejection drug after transplantation, being mechanistically implicated in many cell types, such as naïve CD4^+^T cells, regulatory T cells (Tregs), dendritic cells (DCs), and myeloid-derived suppressor cells (MDSCs) (12). Furthermore, several RAPA derivatives, including everolimus and temsirolimus, are also applied in clinical settings for anticancer or anti-rejection treatment (12, 13). In addition, RAPA can be used with drug-eluting stents for coronary artery disease therapy (14).

Emerging studies have reported that RAPA in systemic administration is also an effective and potent immunosuppressive agent against corneal allograft rejection (15–19). However, its high water insolubility and low bioavailability have greatly hindered its widespread clinical application in ophthalmology (20, 21). Moreover, the mechanisms of RAPA against corneal allograft rejection remain largely unknown. Therefore, improving the water solubility and corneal permeability of RAPA, as well as more clearly elucidating the mechanisms involved in corneal allograft rejection, are crucial for its clinical usage.

In this study, we evaluated the effect of nano–micelle-modified RAPA on corneal allograft rejection and also investigated its mechanism for preventing corneal allograft rejection. Our results using mouse and rabbit corneal-transplantation models clearly show that RAPA nano-micelle could significantly inhibit corneal graft rejection. RAPA could promote recruitment of MDSCs and improve their immunosuppressive function through up-regulation of arginase-1 (Arg-1) and inducible nitric oxidase (iNOS). Our results shed light on future corneal-graft rejection treatment based on RAPA.

## Materials and methods

### Animals

Six-week-old male BALB/C and C57BL/6 mice were purchased from Ji'nan Pengyue Experimental Animal-Breeding Co., Ltd. (Ji'nan, Shandong, China), and New Zealand white rabbits were obtained from Qingdao Kangda Foodstuffs Co., Ltd. (Qingdao, Shandong, China). The use of animals in this study adhered to the ARVO Statement for the Use of Animals in Ophthalmic and Vision Research, and the animal experiments were conducted under pathogen-free conditions in line with institutional animal-care protocols approved by Shandong Eye Institute.

### Murine orthotopic corneal transplantation model

Penetrating keratoplasty (PKP) was performed as previously described (22). In brief, the donor corneas (2 mm in diameter) were excised from C57BL/6 mice and fixed in the recipient graft beds prepared by 1.5-mm trephine with eight interrupted 11-0 nylon sutures (Mani, Inc., Japan) in the central corneas of recipient BALB/c mice. The eyelids were closed after the operation with 8-0 nylon sutures. The eyelid sutures were removed after 3 days, and the corneal grafts sutures were removed 7 days after surgery. Grafts with severe complications, such as infection or hyphemia, were excluded from the study.

### Rabbit high-risk penetrating keratoplasty model

Corneal neovascularization was induced in the right eye of rabbits (male, *n* = 42) using a 5-0 silk suture in each quadrant. Within 2 weeks, the cornea was strongly vascularized, with vessels having grown at least 4 mm into the cornea in three or more quadrants. Afterward, the sutures were removed and PKP was performed.

The surgical techniques for PKP have been previously described (20). Briefly, anesthesia was achieved by intramuscular injection of 25 mg/kg ketamine and 25 mg/kg chlorpromazine. A 7.75-mm trephine was used to produce a corneal button graft, and the recipient cornea was prepared similarly with a 7.5-mm biopsy punch. The donor grafts were then placed centrally into the vascularized bed with a 10-0 monofilament nylon suture (Mani, Inc., Japan). Heparin (1,000 U/ml) was used topically to prevent aqueous clotting during the operation. A 0.3% ofloxacin eye ointment (Santen, Osaka, Japan) was applied at the end of the procedure and once daily for three consecutive days. All PKPs were performed by the same surgeon, who had extensive experience in this technique.

### RAPA nano-micelle eyedrops preparation and encapsulation-efficiency assessment

The preparation of RAPA-loaded PVCL-PVA-PEG nano-micelles has previously been described (23). Briefly, 900.0 mg of PVCL-PVA-PEG, 50.0 mg of RAPA, and 425.0 mg of glucose were co-dissolved in 2 ml of dehydrated ethanol in a round-bottomed flask. The solvent was evaporated under reduced pressure at 40°C to obtain a thin layer of uniform film on the wall of the flask. The residual film was then hydrated with 10.0 ml of phosphate buffer saline (PBS) under moderate shaking. Under these conditions, the amphiphilic PVCL-PVA-PEG copolymer self-assembled into nano-micelles, and RAPA was encapsulated into the micelles. The nano-micelles were filtered through a 0.22 μm filter to obtain sterile formulations and were diluted with PBS to obtain 0.1% (mg/ml) RAPA nano-micelle ophthalmic solution.

The encapsulation efficiency was evaluated as previously described (23). Briefly, 100 μl of RAPA nano-micelle solution was added to 900 μl of methanol and vortexed for 2min. Subsequently, a further 10-fold dilution with methanol was tested by high performance liquid chromatography (HPLC). The encapsulation efficiency was expressed as the ratio of the detected to the added drug amount.

### Ocular-irritation test

The ocular-irritation profiles of the RAPA nano-micelle ophthalmic solution were evaluated in New Zealand rabbits using a modified Draize eye test (23). The rabbits (*n* = 10/group) were used to evaluate ocular tolerance. The 0.1% RAPA nano-micelle ophthalmic solution was instilled into the right eye for 30 min for a total of 13 times, and the left eye was instilled with PBS as a control. Clinical signs were evaluated before the test and at 1, 6, and 24 h after the last instillation. The rabbits' eyes were inspected with a slit-lamp microscope at each time point, and 2% sodium fluorescein was used to detect subtle corneal epithelial damage under cobalt-blue light. Irritation was scored using the Draize scoring system, and the criteria were as follows: non-irritant (scores: 0–3); slight irritant (scores: 4–8); moderate irritant (scores: 9–12); severe irritant (scores: 13–16).

### Treatment

Recipient mice (*n* = 20/group) and rabbits (*n* = 10/group) were treated topically with 0.1% RAPA nano-micelle ophthalmic solution three times per day from postoperative day 1 to day 30 and 140, respectively. To deplete MDSCs *in vivo*, RAPA-treated recipients received 6 μg of anti-Gr-1 antibody (RB6-8C5, R&D Systems) or anti-Ly6G antibody (1A8, R&D Systems) through subconjunctival injection on postoperative days 4, 9, and 14. An isotype-matched rat IgG2b antibody was used as the control (BD Biosciences). To inhibit iNOS activity, RAPA-administrated recipient mice (*n* = 9–13 per group) received aminoguanidine hydrochloride (Selleck Chemicals) (200 mg/kg) by gavage on postoperative days 3, 6, and 9.

### Isolation of MDSCs

Ly6G^+^/Ly6C^low^/CD11b^+^ and Ly6G^−^/Ly6C ^high^/CD11b^+^ MDSCs (G-MDSCs and M-MDSCs, respectively) were isolated from single-cell suspensions prepared from the spleens of the recipients. The cells were isolated by magnetically activated cell sorting (MACS) using a mouse myeloid-derived suppressor cell isolation kit (Miltenyi Biotec, Auburn, CA) according to the protocol provided by the manufacturer. The purity of the G-MDSC and M-MDSC populations was >90% as determined by a FACSCalibur.

### Flow cytometry analysis

Nonspecific mAb binding to the recovered splenocytes was prevented by incubation with anti-CD16/CD32 (eBioscience, San Diego, CA), and the cells were then stained with anti-mouse antibodies against PE-Gr-1 (clone: RB6-8C5) (eBioscience, San Diego, CA), FITC-CD11b (M1/70). Labeled cells were acquired using a FACSCalibur flow cytometer (BD Bioscience) and analyzed using WinMDI 2.9 software.

### Adoptive transfer of MDSCs

G-MDSCs and M-MDSCs were isolated from mice treated with RAPA or PBS following keratoplasty, and 2 × 10^6^ cells of each phenotype suspended in 6 μl PBS were transferred to the recipients' right eyes (*n* = 8–10 per group) via subconjunctival injection on postoperative day 2. Pinholes were ligated using purse-string sutures to prevent leakage.

### CD4^+^T cells proliferation assay

Splenocytes, which were used as responder cells, were derived from naive BALB/C mice and labeled with carboxyfluorescein succinimidyl ester (CFSE) (5 μM; Invitrogen). The CFSE-labeled splenocytes (2 × 10^5^ cells/well) were cocultured with purified G-MDSCs and M-MDSCs, and stimulated with anti-mouse CD28 (0.5 μg/ml, eBioscience) and anti-mouse CD3e (10 μg/ml, eBioscience) in a flat-bottomed 96-well plate. The ratio of splenocytes to MDSCs was 2:1. *N*^G^-hydroxy-L-arginine (an inhibitor of arginase, Selleck Chemicals) or L-NMMA (an inhibitor of NOS activity, Selleck Chemicals) were added to the wells at a final concentration of 500 μM. After 3 days of co-culture, the proliferation of CD4^+^ T cells was evaluated by flow cytometry and analyzed as follows:

T-cell inhibition (%) = (1-proliferation rate with MDSC/proliferation rate without MDSC) × 100%

### Immunofluorescent microscopy

Allografts from the transplanted groups (*n* = 5/group) were collected at 3 weeks. Six- to eight-micrometer serial frozen sections of each eye were prepared for immunohistological examination. PE-anti-CD4 (eBioscience), PE-anti-Ly6G (eBioscience), FITC-anti-F4/80 (eBioscience), and PE-anti-CD11C (eBioscience) were used as primary antibodies for immunofluorescence staining. DAPI staining was used to reveal all cells in the section.

### Quantitative RT-PCR

Total RNA was extracted from MDSCs or corneal-allograft samples (*n* = 5/group) using TRIzol reagent (Invitrogen, Carlsbad, CA, USA) and was reverse-transcribed using reverse transcriptase (Toyobo, Osaka, Japan). Quantitative real-time PCR analysis was performed on an ABI Prism 7500 (Applied Biosystems, Foster City, CA, USA) using SYBR Green mix (Toyobo, Osaka, Japan). Data were analyzed by comparative threshold method(2^−ΔΔ*CT*^) and normalized using GAPDH as internal control. The primer sequences were listed in Supplementary Table 1.

### Western blot

Corneal allografts and MDSCs were lysed in RIPA buffer (Beyotime, Beijing, China) with a protease inhibitor cocktail (Millipore), respectively. Western blot was performed as described previously (24). The membranes were probed with anti-GAPDH (KC-5G5, Kangchen) and anti-iNOS (ab15323, Abcam) primary antibodies and subsequently reacted with HRP-conjugated secondary antibodies (Pierce, 1:3,000), respectively.

### Enzyme-linked immunosorbent assay (ELISA)

Corneal-graft samples were dissected from three groups (*n* = 5/group) at 20 days and were then homogenized and placed in RIPA lysis buffer (Beyotime, Beijing, China) and Halt protease inhibitor (Thermo Scientific, USA). The solutions were centrifuged at 10,000 g for 5 min. The resulting protein supernatant was analyzed for total concentration using an enhanced BCA protein-assay kit (Beyotime, Beijing, China) according to the manufacturer's instructions. ELISA was then conducted in triplicate on protein samples using mouse ELISA kits (Qiaoyi, Anhui, China) according to the manufacturer's instructions.

### Statistical analysis

All experiments were performed at least three times, and the data were presented as mean ± SD. A two-tailed Student's *t*-test was used for comparisons between two groups, and one-way analysis of variance (ANOVA) was employed for comparisons among more than two groups. The Kaplan-Meier method was applied to evaluate the cumulative survival rate of corneal allografts. A value of *p* < 0.05 was considered statistically significant.

## Results

### RAPA nano-micelle displays no ocular irritation

As shown in Supplementary Figure 1, RAPA-loaded nano-micelles were transparent, slightly opalescent, and off white when compared to phosphate buffer saline (PBS). The encapsulation efficiency was 96.04 ± 0.12%.

The ocular irritation of the RAPA nano-micelle was evaluated in New Zealand rabbits using a modified Draize eye test. The scores describing irritation of the cornea, conjunctiva, and iris were assessed, and grades for corneal and conjunctival staining were recorded. As shown in Supplementary Figure 2, no conjunctival congestion, corneal opacity, or iris-inflammatory exudation was observed in the RAPA nano–micelle-treated group. The score of the RAPA nano-micelle group met the standard of non-irritation, and no significant difference was observed between the nano-micelle group and the PBS group (Supplementary Figure 2A). Fluorescein staining indicated that there were no ulcers, scratches, or defects in the corneal epithelium at the end of the modified Draize test (Supplementary Figure 2B). Moreover, no obvious pathological changes were found at the end of the modified Draize test by hematoxylin-eosin (H-E) staining (Supplementary Figure 2C). These results verified that RAPA nano-micelle was relatively safe for ocular application.

### RAPA nano-micelle ophthalmic solution significantly delays corneal allograft rejection

**Figure 1:**
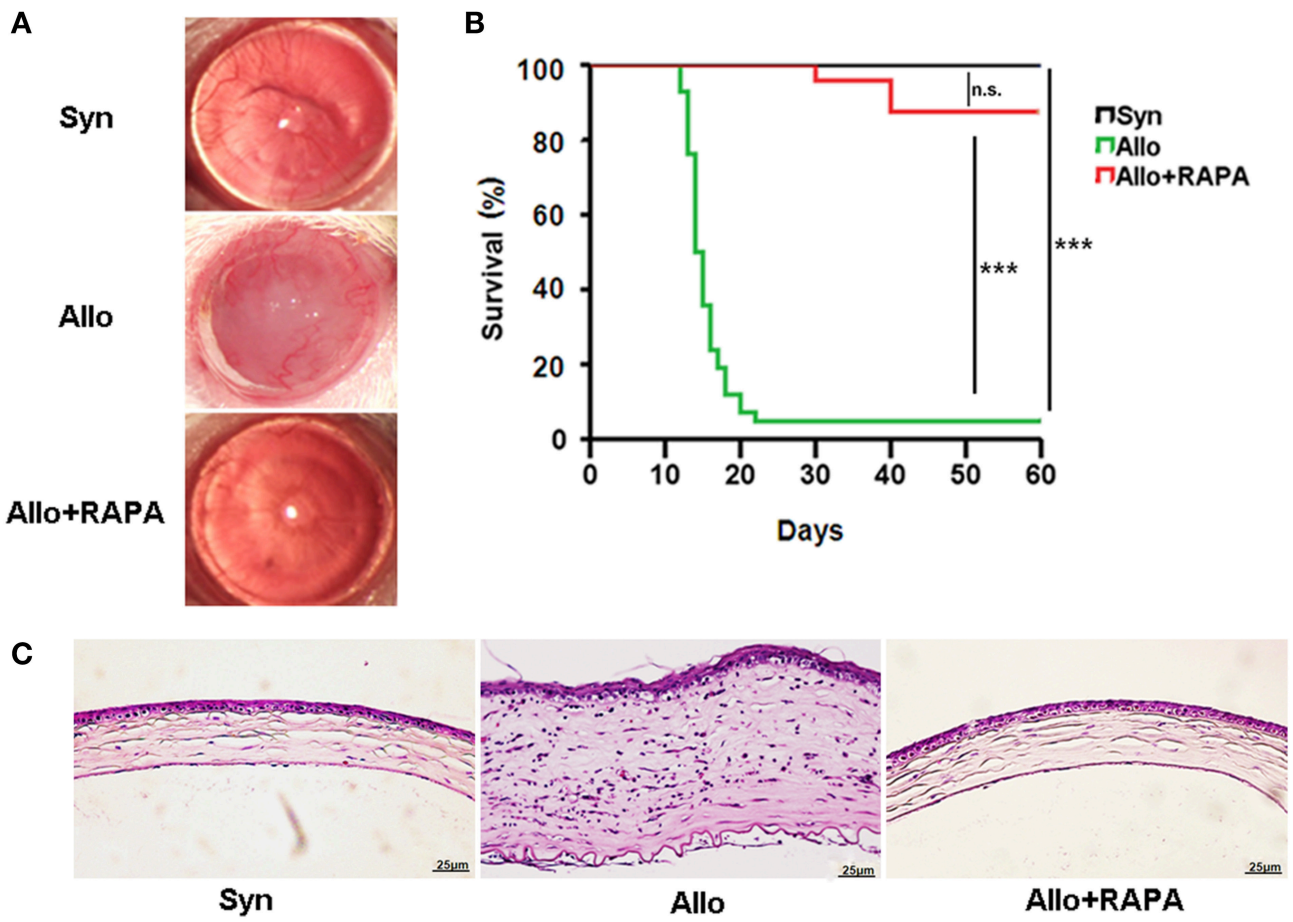
RAPA nano-micelle ophthalmic solution significantly prolongs the survival of corneal allograft in mouse models. **(A)** The representative photographs of corneal grafts in different groups. **(B)** Corneal allografts survival rate (*n* = 20/ group). **(C)** Histological changes in corneal allografts on postoperative day 20 by H-E staining. ^***^p < 0.001, n.s., no significance. Scale bar = 25 μm.

**Figure 2:**
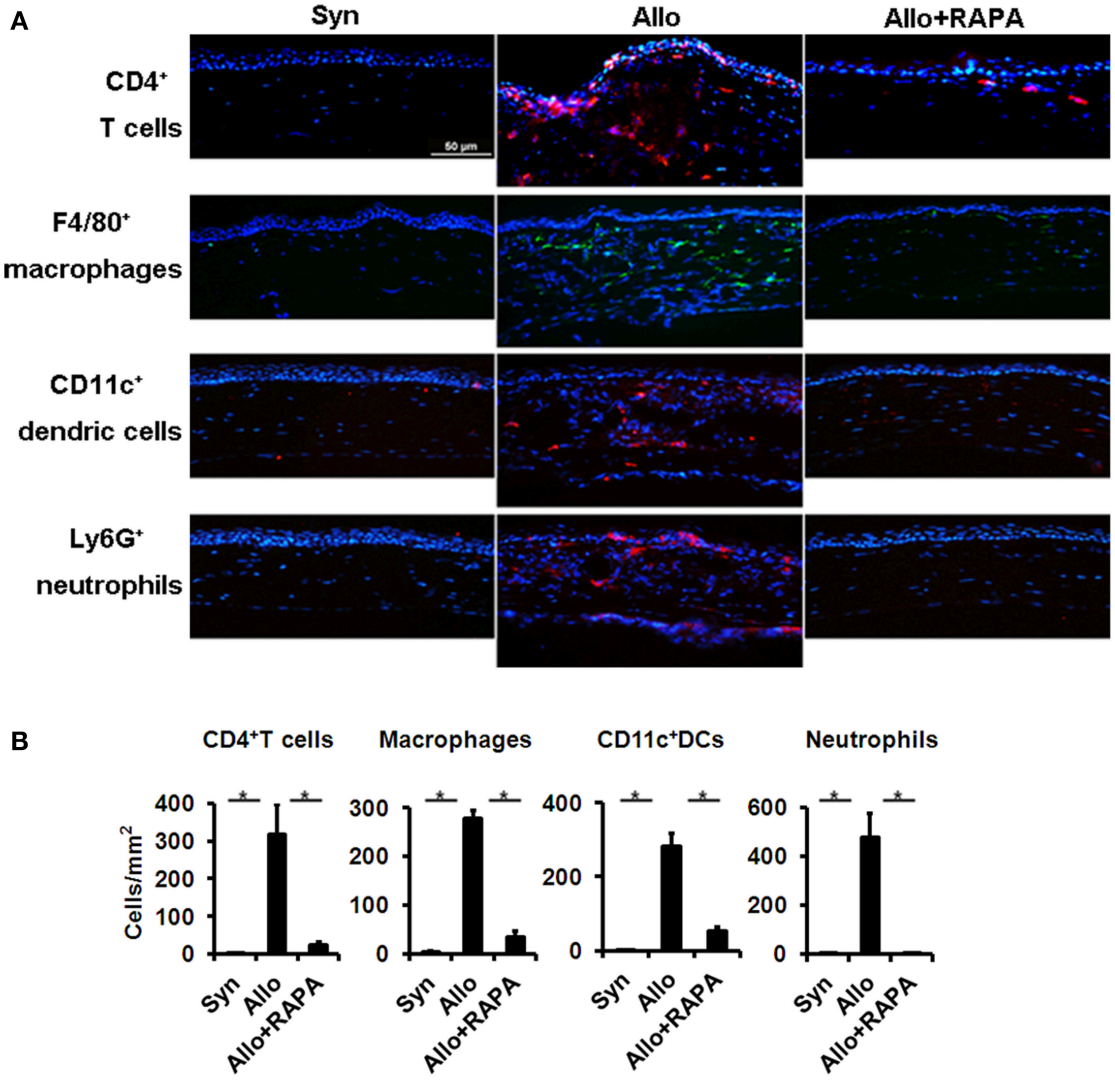
RAPA nano-micelle inhibits infiltration of inflammatory cells in corneal allografts. **(A)** Immunofluorescent staining shows CD4^+^T cells, macrophages, dendritic cells, and neutrophils infiltration in corneal allografts. **(B)** Quantitative data of infiltrated CD4^+^T cells, macrophages, dendritic cells, and neutrophils as in **(A)** (*n* = 3 per group). ^*^p < 0.05. Scale bar = 50 μm.

**Figure 3:**
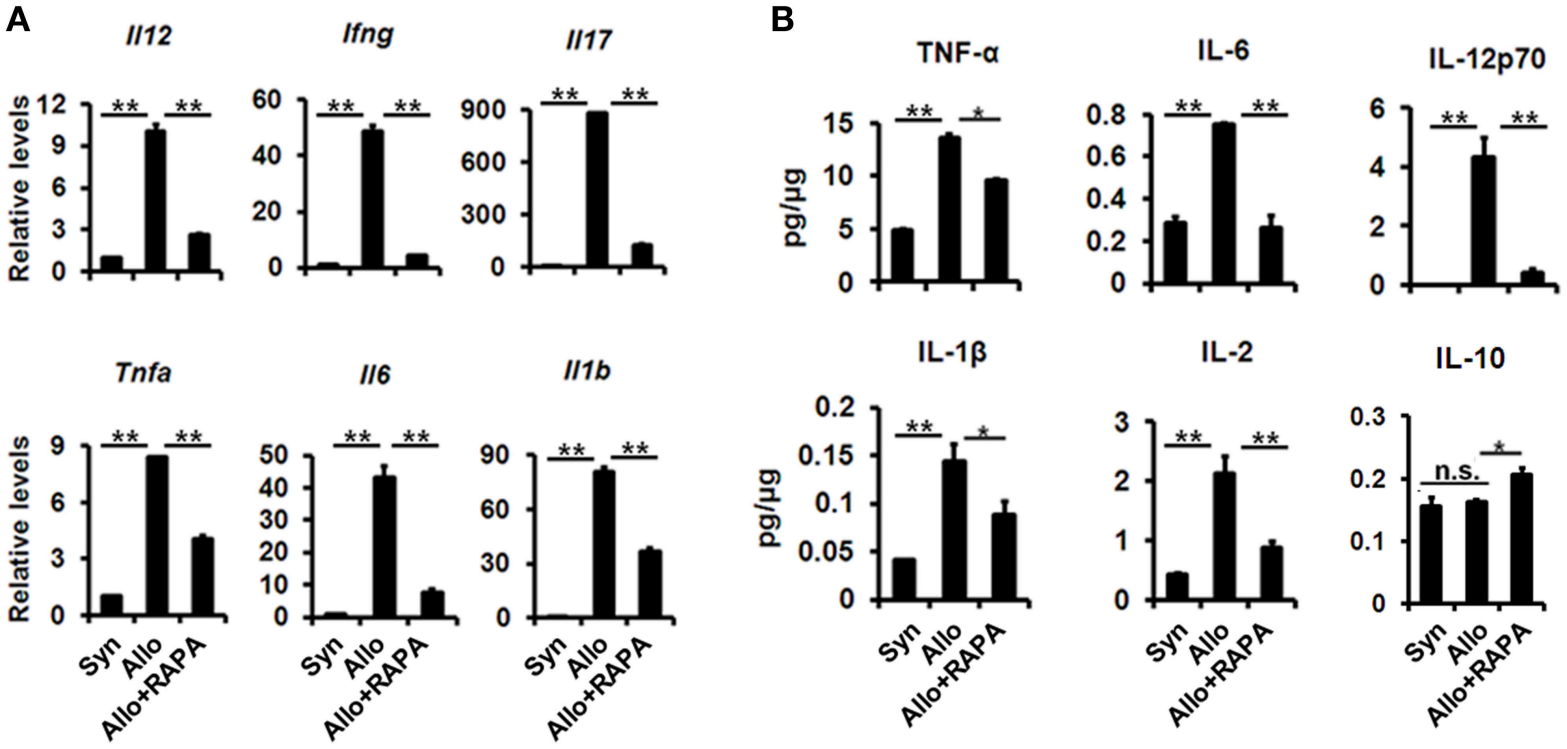
Improvement of corneal allografts rejection in RAPA nano-micelle treated mouse is associated with decreased expression of pro-inflammatory cytokines. **(A)** Real-time PCR for IL-12, IFN-γ, IL-17, TNF-α, IL-6, and IL-1β mRNA expression in corneal allografts on postoperative day 20 (*n* = 5/group). **(B)** The protein expression of TNF-α, IL-6, IL-12p70, IL-1β, IL-2, and IL-10 in corneal allografts on postoperative day 20 by ELISA (*n* = 5/group). ^*^p < 0.05, ^**^p < 0.01, n.s., no significance.

To investigate the effect of RAPA nano-micelle ophthalmic solution on corneal allograft rejection, we transplanted the cornea from C57BL/6 mice into BALB/c mice, and then administered RAPA nano-micelle ophthalmic solution. The corneal allografts in recipients without RAPA nano-micelle treatment survived for less than 30 days, with pronounced corneal opacity and corneal edema (Figures 1A,B). However, the allografts in mice instilled with RAPA nano-micelle ophthalmic solution survived for more than 60 days (Figures 1A,B). Histology and immunofluorescence analysis showed that corneal allografts from recipient mice without RAPA nano-micelle developed severe allograft rejection, with much more infiltration of CD4^+^T cells, CD11c^+^ dendritic cells, F4/80^+^ macrophages, and Ly6G^+^ neutrophils (Figures 1C, 2) and a marked up-regulation of pro-inflammatory cytokines, including tumor necrosis factor-alpha (TNFα), interferon-gamma (IFN-γ), interleukin-6 (IL-6), IL-2, and IL-1β, leading to a lower graft survival (Figures 3A,B). In contrast, corneal allografts from mice treated with RAPA nano-micelle ophthalmic solution showed less severe allograft rejection, with less inflammatory infiltration, decreased expression of pro-inflammatory cytokines such as TNFα, IL-6, IL-12p70, and IL-2, and elevated levels of IL-10, resulting in a remarkable improvement in allograft survival (Figures 2, 3A,B). Furthermore, we also found that both RAPA nano-micelle ophthalmic solution and conventional RAPA eye drops could significantly prolong the survival of corneal allograft, but RAPA nano-micelle ophthalmic solution is far more effective than conventional RAPA eye drops in preventing corneal allograft rejection (Supplementary Figure 3).Thus, RAPA nano-micelle ophthalmic solution significantly prolonged the survival of corneal allografts, with an anti-rejection advantage over conventional RAPA eye drops.

### Increased MDSC accumulation in RAPA-treated mice following corneal transplantation

**Figure 4:**
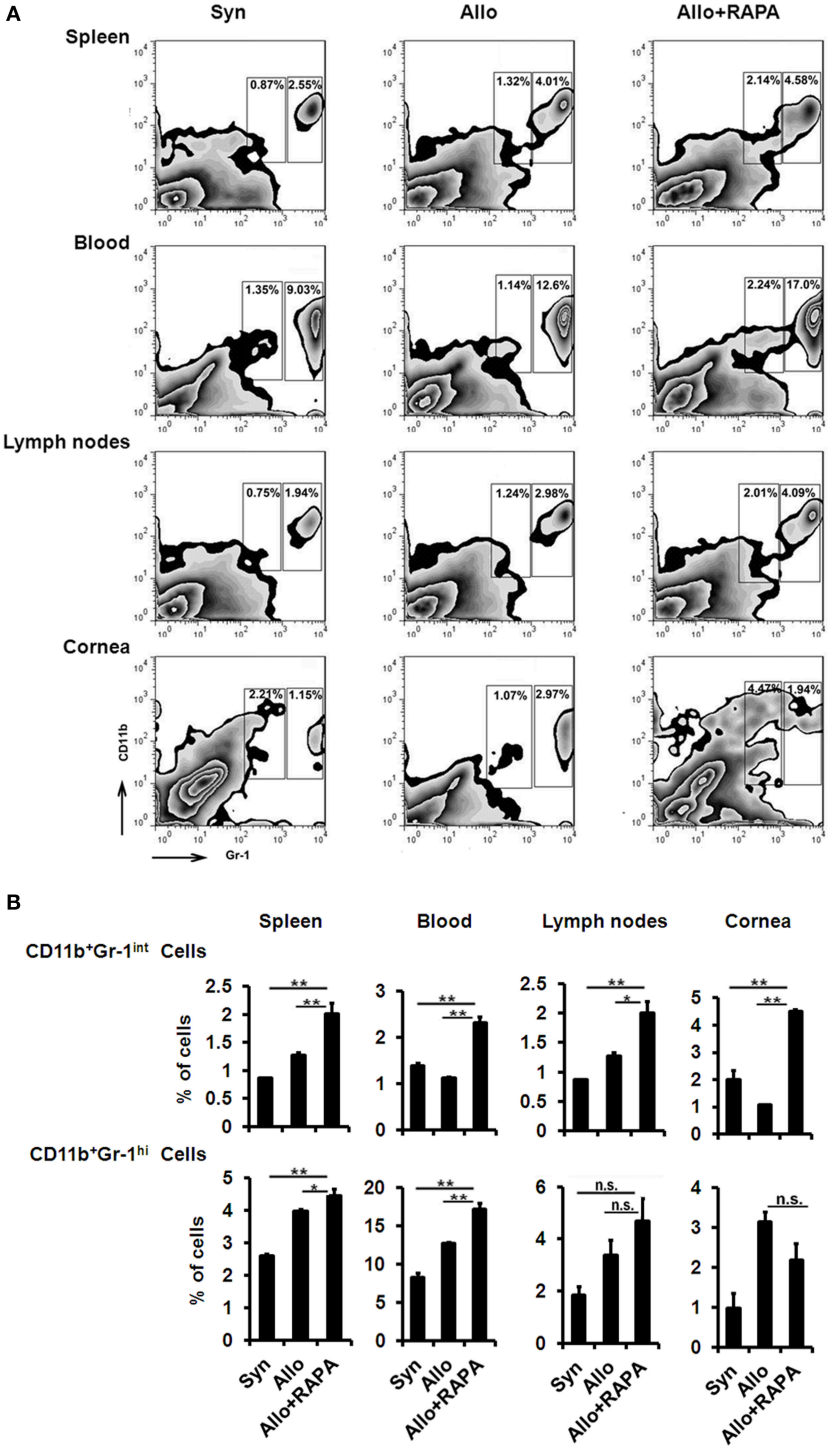
Increased MDSCs accumulation in RAPA-treated mice following corneal allograft transplantation. **(A)** Representative flow cytometric plots of CD11b^+^Gr-1^+^MDSCs in spleen, blood, cervical lymph nodes and corneal allografts by two-color flow cytometry from Syn, Allo, and Allo + RAPA nano-micelle group, respectively. **(B)** Quantitative data of MDSCs on two-color flow cytometry analysis as in A (*n* = 5/group). ^*^p < 0.05, ^**^p < 0.01, n.s., no significance.

It has been reported that MDSCs are involved in the pathogenesis of corneal allograft rejection (25–27) and that RAPA prolongs cardiac-allograft survival and alleviates acute kidney injury by inducing MDSC recruitment and strengthening their immunosuppressive activity (28, 29). Based on these findings, we investigated whether MDSCs are associated with the RAPA-induced anti-rejection effect in corneal-transplantation models. We found that the percentage of Gr1^int^CD11b^+^MDSCs in the corneal allografts, cervical lymph nodes, blood, and spleens of RAPA-treated mice was much more significantly increased than in identical samples from recipients without RAPA treatment (Figures 4A,B). However, the frequency of Gr1^hi^CD11b^+^cells, another cluster of MDSCs, in the blood and spleens of the recipients following administration of RAPA was higher than in samples from mice without RAPA treatment, while the frequency increases of Gr1^hi^CD11b^+^cells in corneal allografts and cervical lymph nodes from RAPA-administrated recipient mice displayed no statistical difference as compared to untreated recipients (Figures 4A,B).

Gr-1^high^ CD11b^+^ cells represented mostly by G-MDSCs, featured with Ly6G^+^/Ly6C^low^/CD11b^+^ phenotype, and Gr-1^int^ CD11b^+^ cells mainly comprised M-MDSCs, characterized by Ly6G^−^/Ly6C^high^/CD11b^+^ phenotype (30). In the following experiment, we found the increased transcriptional expression of CXCR2 in RAPA-treated MDSCs (Supplementary Figure 4A), which regulates the migration of MDSCs. Further investigations revealed the elevated transcriptional expression of CXCR2 in M-MDSCs, but not in G-MDSCs (Supplementary Figure 4B). The differential expression of CXCR2 probably lead to the decreased infiltration of Gr-1^high^ CD11b^+^ cells (Ly6G+ cells), which was consistent with the results reported by Zhang et al. (31). Overall, RAPA nano-micelle eyedrops modulated the hosts' immune response, which allowed Gr-1^int^CD11b^+^cells to accumulate following corneal transplantation.

### The anti-rejection effect of RAPA nano-micelle ophthalmic solution depends on MDSCs

**Figure 5:**
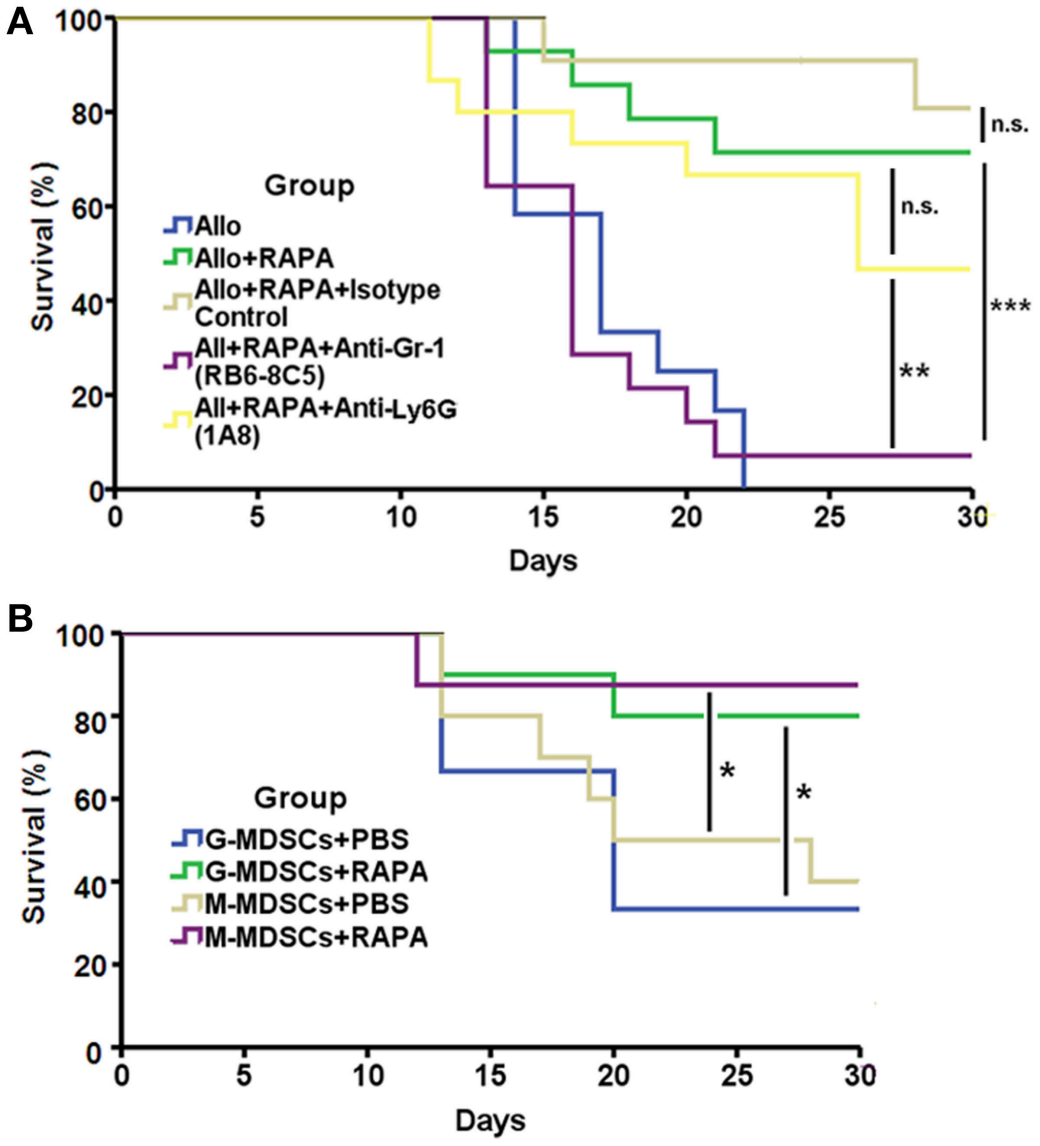
The anti-rejection effect of RAPA nano-micelle depends on MDSCs. **(A)** Survival of allografts transplanted into allogeneic RAPA nano-micelle treated recipients, treated with anti-Gr-1, anti-Ly6G, or rat IgG (isotype control) antibody (6 μg/mouse, subconjunctival injection, on postoperative day4, 9, and 14) (*n* = 11–14 per group). **(B)** Graft survival in the allogeneic recipient mice underwent adoptive transfer of 2 × 10^6^ MDSCs isolated from mice treated with RAPA or PBS following keratoplasty through conjunctival injection, respectively (*n* = 8–10 per group). ^*^p < 0.05, ^**^p < 0.01, ^***^p < 0.001, n.s., no significance.

To confirm whether the protective role of RAPA nano-micelle depended on MDSCs, the recipients additionally received 6 μg of anti-Gr-1 antibody or isotype antibody through subconjunctival injection on postoperative days 4, 9, and 14. We found that corneal-allograft survival time was significantly reduced in recipient mice treated with anti-Gr-1 antibody (RB6-8C5), which blocks both Ly6G (Gr-1^high^ subtype) and Ly6C (Gr-1^int^ subtype) populations (Figure 5A). To know which MDSC subpopulation is responsible for the effects on corneal allotransplants, we further blocked Ly6G population using specific anti-Ly6G antibody (1A8). The results showed that the survival time in anti-Ly6G antibody treated group was also reduced compared with RAPA nano-micelle treated group, but their difference had no statistical significance (Figure 5A). The results revealed that CD11b^+^Gr-1^int^ cells played important roles in RAPA-induced anti-rejection effect.

We also investigated the effect of RAPA on corneal-allograft survival through adoptive transfer of MDSCs. Fully allogeneic corneal allografts were temporarily accepted by mice that received 2 × 10^6^ G- or M-MDSCs isolated from RAPA-administrated mice via subconjunctival injection on day 2. Control mice underwent adoptive transfer of MDSCs from mice treated with PBS. The results revealed that MDSCs pre-treated with RAPA prolonged corneal-allograft survival to a greater extent than did cells treated with PBS (Figure 5B). Taken together, these data support our findings that the anti-rejection effect of RAPA nano-micelle relies at least partially on MDSCs, especially CD11b^+^Gr-1^int^ cells.

### MDSCs derived from mice treated with RAPA nano-micelle ophthalmic solution show increased immunosuppressive activity through iNOs and ARG-1 up-regulation

**Figure 6:**
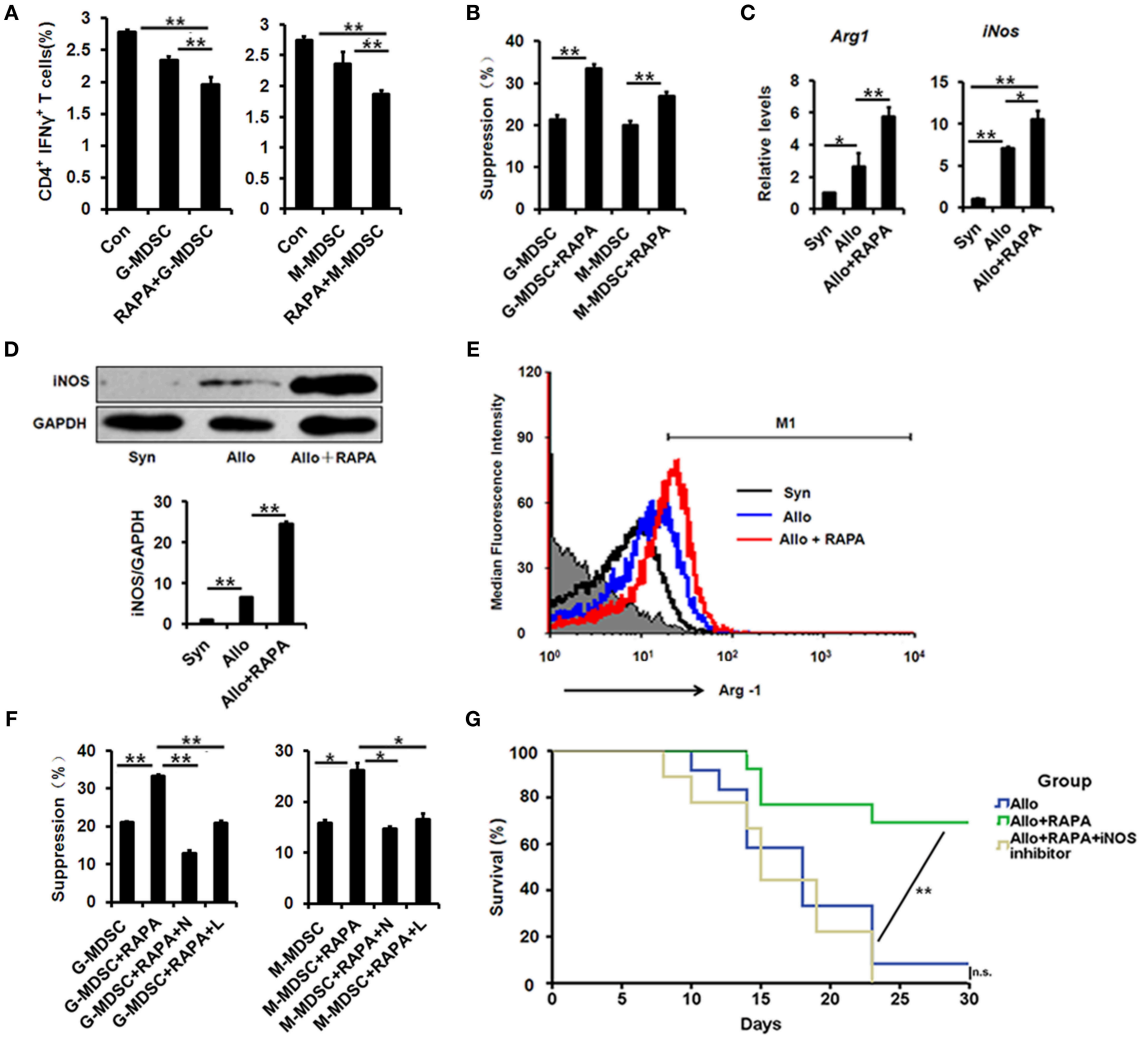
MDSCs derived from RAPA nano-micelle ophthalmic solution treated mice show increased immunosuppressive activity through iNOS and Arg-1 upregulation. **(A)** The representative histograms showing the % suppression of MDSCs on Th1 cells proliferation under different conditions, respectively. **(B)** The representative histograms showing the % suppression of MDSCs on naïve CD4^+^ T cells proliferation under different conditions, respectively **(C)** The real-time PCR for iNOS and Arg-1 mRNA expression in splenic MDSCs isolated from Syn, Allo, and Allo+RAPA nano-micelle group, respectively. **(D)** The expression of iNOS by western blot in splenic MDSCs isolated from Syn, Allo, and Allo+RAPA nano-micelle group, respectively. **(E)** Quantitative analysis of Arg-1 expression using flow cytometry in splenic MDSCs isolated from Syn, Allo, and Allo+RAPA nano-micelle group, respectively. **(F)** The representative histograms showing the % suppression of MDSCs under different conditions. **(G)** Graft survival in allogeneic RAPA nano-micelle administrated recipients, treated with aminoguanidine hydrochloride (200 mg/kg by gavage on postoperative day 3, 6, and 9) (*n* = 9–13 per group). Values are shown as mean ± SD. N, *N*^G^-Hydroxy-L-arginine (an inhibitor of Arg-1); L, L-NMMA (an inhibitor of NOS). ^*^p < 0.05, ^**^p < 0.01.

To examine whether MDSCs from mice treated with RAPA nano-micelle showed increased suppressive activity, two subpopulations (Ly6G^+^/Ly6C^low^/CD11b^+^ cells for G-MDSCs and Ly6G^−^/Ly6C^high^/CD11b^+^ cells for M-MDSCs) were isolated and separately co-cultured with splenocytes *in vitro*. Compared with MDSCs isolated from the untreated group, G-MDSCs and M-MDSCs derived from RAPA-instilled recipients more significantly inhibited the proliferation of Th1 cells and naïve CD4^+^ T cells *in vitro* (Figures 6A,B). These results demonstrate that MDSCs isolated from RAPA-administered recipient mice display increased immunosuppressive activity.

Accumulated evidence indicated that inducible nitric oxide synthase (iNOS) and arginase-1 (Arg-1) were implicated in immunosuppressive regulation in MDSCs (29, 32). Therefore, we investigated whether iNOS and Arg-1 were also engaged in the functional improvement of MDSCs from RAPA-treated mice. Compared with untreated MDSCs, increased transcriptional levels of iNOS and Arg-1 were detected in RAPA-treated MDSCs using quantitative real-time PCR (Figure 6C), and their elevated protein levels were confirmed using Western blot and flow-cytometric analysis, respectively (Figures 6D,E). The results suggest that their enzymatic activity contributes to their functional improvement. Thus, the following experiments were performed to test whether their enzymatic activity correlated with their functional improvement. The enhanced immunosuppressive ability was observed in RAPA-treated M-MDSCs and G-MDSCs, while their immunosuppressive activity was reversed both in M-MDSCs and G-MDSCs following treatment with ^*N*^G-hydroxy-L-arginine (an inhibitor of Arg-1) (Figure 6F). The results revealed a positive correlation between Arg-1 levels and immunosuppressive activity of MDSCs induced by RAPA.

We further investigated the roles of iNOS in different MDSC subpopulations in details. We found that RAPA significantly augmented the expression of iNOS both in M-MDSCs and G-MDSCs (Supplementary Figure 5), suggested the roles of iNOS in RAPA-mediating immunosuppressive activity. Moreover, L-NMMA, an inhibitor of NOS, could also significantly destroy their immunosuppressive activity induced by RAPA both in M-MDSCs and G-MDSCs (Figure 6F). In addition, inhibition of iNOS pharmacologically using aminoguanidine hydrochloride (an inhibitor of iNOS; 200 mg/kg via gavage on postoperative days 3, 6, and 9) also destroyed the anti-rejection effect of RAPA on corneal allografts, which supports our findings that RAPA-loaded nano-micelles prolonged corneal-allograft survival through iNOS (Figure 6G). Collectively, RAPA nano-micelle probably strengthened their immunosuppressive activity by elevated expression of Arg-1 and iNOS, at least partially.

### RAPA nano-micelle ophthalmic solution significantly promotes corneal allograft survival in high-risk corneal transplantation

**Figure 7:**
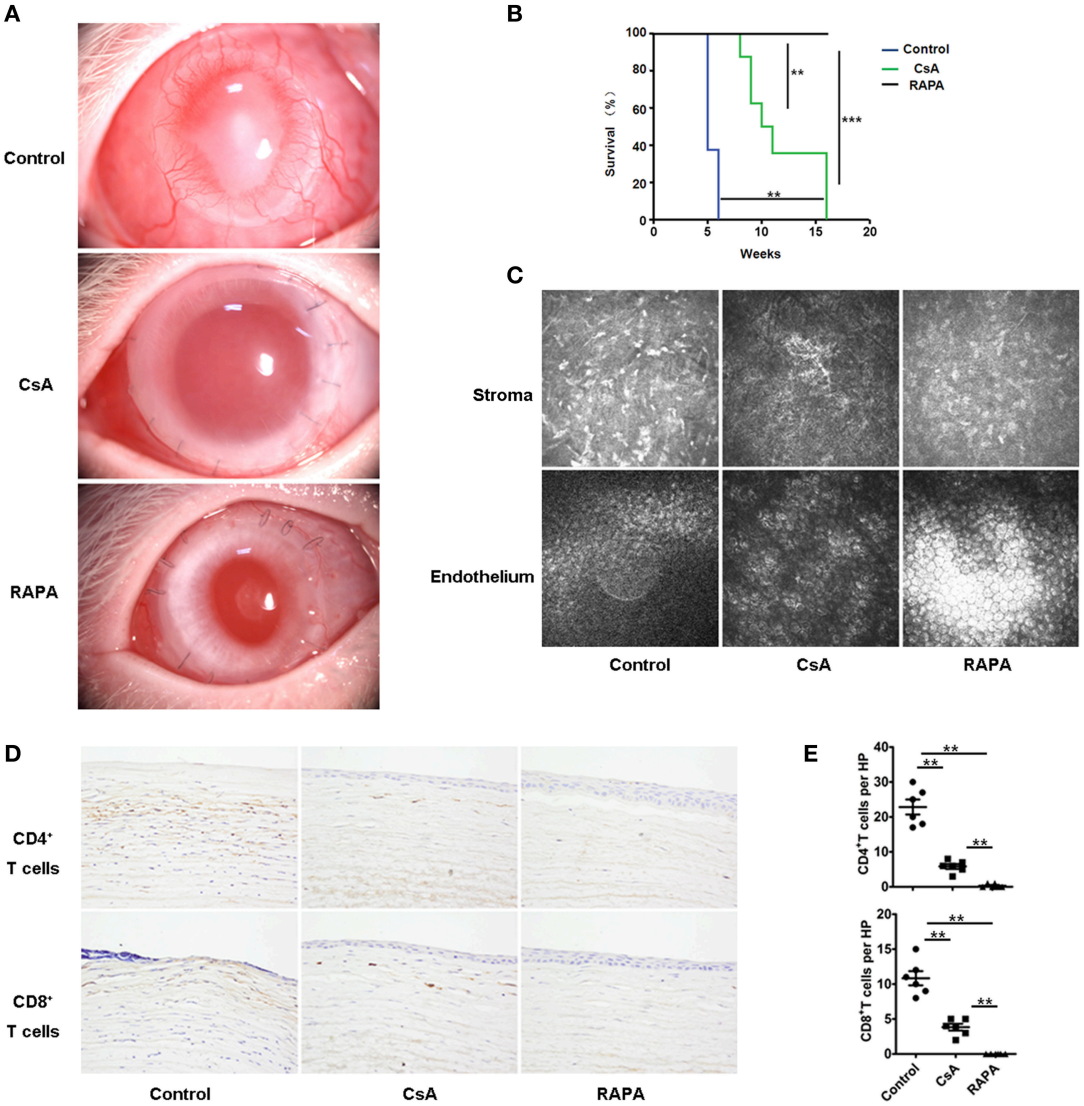
RAPA nano-micelle ophthalmic solution significantly promotes corneal allograft survival in high-risk corneal transplantation. **(A)** The clinical appearance of corneal grafts in different groups. **(B)** Corneal allografts survival rate within 120 days (*n* = 10/group). **(C)** Representative confocal images of corneal stroma and endothelium in different groups (*n* = 5/group). **(D)** Infiltration of CD4^+^T cells and CD8^+^T cells was detected by immunohistochemistry in corneal grafts. **(E)** Quantitative data of CD4^+^T cells and CD8^+^T cells infiltrating the corneal grafts (*n* = 5/group). Data are expressed as the mean ± SD. ^**^p < 0.01, ^***^p < 0.001.

Corneal neovascularization is the greatest risk factor in corneal transplantation. Therefore, we investigated whether RAPA nano-micelle administration also inhibited rejection in rabbit PKP models with corneal neovascularization. Corneal-graft rejection was observed in rabbits without RAPA nano-micelle or cyclosporin A (CsA) eyedrop treatment, presented as obvious corneal opacity and corneal edema with vascularization and thickening (Figures 7A,B). Conversely, RAPA nano-micelle and CsA eyedrops effectively inhibited corneal graft rejection when compared with the control group (Figures 7A,B). However, the RAPA nano-micelle treatment was more effective than CsA eyedrops and featured an increased corneal-graft survival rate, pronounced corneal clarity, and less edema (Figures 7A,B). Confocal microscope images showed much more inflammatory infiltration in stroma and irregular corneal endothelial cells in recipient rabbits without RAPA nano-micelle treatment, while there were fewer inflammatory cells in stroma and many more regular corneal endothelial cells in the endothelium of rabbits following RAPA nano-micelle administration (Figure 7C). Furthermore, less infiltration of CD4^+^T cells and CD8^+^T cells was observed in corneas treated with RAPA nano-micelle than in the untreated group or the CsA-eyedrops-treated group (Figures 7D,E). Collectively, RAPA nano-micelle effectively alleviated corneal graft rejection in rabbit corneal transplantation with corneal vascularization, and its effect was overwhelmingly superior to that of the CsA eyedrops.

## Discussion

Although keratoplasty is a widely practiced surgical operation to save visual function, the success rate of high-risk corneal grafts is as low as 20–40% (7, 8). Therefore, ani-rejection therapy after keratoplasty is of paramount importance for preserving the vision of patients, especially in high-risk cases. However, due to their low solubility, unfavorable stability, or side effects, traditional immunosuppressants are unsatisfactory for clinical application (33). Although emerging evidence had demonstrated that RAPA was a novel immunosuppressive agent for prevention of corneal-allograft rejection (15–19), its water insolubility and the existence of a blood/eye barrier has restricted its clinical application. In this study, we successfully developed RAPA-loaded nano-micelles and found that they effectively inhibited corneal grafts rejection and prolonged the survival of corneal grafts in recipient mice and rabbits with corneal vascularization. Furthermore, we showed that increased MDSC recruitment and improved immunosuppressive activity through elevated expression of Arg-1 and iNOS followed RAPA treatment, thereby preventing corneal allograft rejection.

Systemic application of RAPA has proven effective for preventing corneal-allograft rejection in animal models and in patients with keratoplasty. However, systemic RAPA administration has been reported to cause some side effects, such as hyperlipidemia, infectious and non-infectious pneumonia, anemia, and lymphocele formation (34, 35). In this regard, topical application of RAPA is conducive to avoiding such adverse effects. Ophthalmic solutions are the most popular drug-delivery system for the management of ocular diseases, especially diseases occurring in the anterior segment (36, 37). However, water insolubility restricts its topical application in ophthalmology. In the present study, we successfully developed an RAPA-loaded nano-micelle and found that a 0.1% RAPA nano-micelle ophthalmic solution significantly alleviated corneal-allograft rejection and prolonged allograft survival in mouse and rabbit models, which shed light on its ophthalmological clinical application. Nevertheless, the concentrations of RAPA in the cornea and aqueous humor require further quantification.

Ocular irritancy is a key parameter for evaluating the safety of any substance that is supposed to be administered to the ocular surface. The Draize test is a widely used classic test of ocular irritancy (38). In our study, we used a modified Draize test to determine the ocular irritancy of RAPA nano-micelle and found that the RAPA nano-micelle ophthalmic solution caused no signs of edema, redness, swelling, ulceration, conjunctival congestion, or opacity during the follow-up, based on the irritancy criteria. However, the Draize test remains controversial because of anatomical and biochemical differences between rabbit and human eyes as well as because of ethical concerns regarding the use of animals (36). Therefore, alternative approaches are required to supplement the Draize test. For example, the use of human corneal cell lines is a good alternative approach for testing chemical-induced irritancy (39). Although a modified Draize test preliminarily verified the safety of the RAPA nano-micelle, further safety evaluations are indispensable for future clinical applications.

Systemic administration of RAPA was proven effective for preventing corneal allograft rejection in animal models. However, research on the mechanism underlying its effect focused mainly on Tregs, and the other molecular mechanisms of RAPA implicated in anti-rejection treatment were largely unknown. Growing evidence indicated that adoptive transfer MDSCs protected islet cells against rejection as well as preventing corneal allograft rejection (25, 26, 40). Some research also demonstrated that RAPA could significantly prolong cardiac-allograft survival and protect against murine immunological hepatic injury or acute kidney injury by inducing MDSCs (28, 29, 31). However, it was largely unknown whether the therapeutic effect of RAPA on corneal transplantation relies on MDSCs. In the present study, we showed that RAPA treatment potentiates the recruitment of MDSCs in murine corneal-transplantation models. Furthermore, MDSCs from RAPA-treated mice showed improved immunosuppressive activity, which could significantly inhibit CD4^+^T-cell proliferation.

There are several mechanisms by which RAPA nano-micelle prevents corneal allograft rejection through regulating MDSCs. One is that RAPA nano-micelle inhibited corneal allograft rejection through regulating MDSC recruitment. Several studies indicated that RAPA could prolong cardiac-allograft survival and protect against murine immunological hepatic injury by promoting CD11b^+^Gr-1^int^ cells recruitment (28, 41). Likewise, Choi et al. reported that CD11b^+^Gr-1^int^ cells improved corneal allograft survival (27, 42). Consistent with these findings, our study revealed that RAPA nano-micelle significantly delayed corneal allograft rejection by promoting CD11b^+^Gr-1^int^ cells recruitment. Another potential mechanism of RAPA is immunosuppressive activity induced by Arg-1 and iNOS. Accumulative evidence indicated that M-MDSCs produce Arg-1 and iNOS, both of which suppress T-cell proliferation, while G-MDSCs inhibit T-cell proliferation by producing Arg-1 and ROS (42, 43). In this study, however, our results showed an increased expression of Arg-1 and iNOS in RAPA-treated MDSCs, and blocking their enzymatic activity effectively precluded the immunosuppressive function of M-MDSCs and G-MDSCs. These results are in line with other studies in favor of the notion that RAPA strengthened their immunosuppressive activity of M-MDSCs and G-MDSCs (29). In addition, we also found that the infiltrated Gr1^hi^CD11b^+^cells in RAPA-treated corneal allografts showed decreased frequencies but enhanced immunosuppressive function, which suggested that Gr1^hi^CD11b^+^cells could also partially contributed to RAPA-induced long-term graft acceptance in corneal allotransplants. Taken together, the data suggest that the functional improvement of MDSCs through RAPA treatment probably relies on increased expressions of Arg-1 and iNOS.

The limitations of the present study must also be acknowledged. First, the chemical properties and stability of RAPA nano-micelles should be investigated. Second, given that the Draize test remains controversial for assessing ocular irritancy; alternative approaches should be adopted to further evaluate ocular irritancy. Furthermore, although our results determined the anti-rejection effect within 2 months, the anti-rejection effect of RAPA nano-micelles for longer periods requires further investigation. Finally, a growing number of evidence indicated that RAPA involves the generation of Tregs and that MDSCs have their own ability to regulate Tregs (44, 45). Therefore, Tregs in our setting needs to be evaluated, further studies are also needed to investigate whether Tregs participate in MDSC-mediated tolerance induced by RAPA. Despite these limitations, this is one of the first studies to reveal several key properties of a RAPA nano-micelle ophthalmic solution, including ocular safety and anti-rejection efficacy.

In conclusion, our results suggest that RAPA-loaded micelles significantly blunt immune rejection and prolong corneal-graft survival through regulation of functional MDSCs. Additional insight into the control of MDSCs through RAPA treatment will not only illustrate the molecular principles of MDSC regulation, but also facilitate clinical applications for prevention of corneal-allograft rejection. Future studies are needed to determine in detail its ocular distributions and concentrations and to uncover the mechanism underlying MDSC recruitment and function.

## Ethics statement

All animal experiments were carried out and approved adhered to the guidelines of the animal care and the Shandong Eye Institute Ethics Committee for Animal Experimentation (Approval document No 2012-6, Qingdao, Shandong, China).

## Author contributions

All authors have actively contributed to the study. HG and WS designed the study; YW, LM, XW, HC, SZ, TL, DX and ZL performed the experiments. YD and XgW contributed to the experiments. HG, CW, LM and ZL analyzed the data. HG, WS and CW wrote the manuscript. All authors reviewed the manuscript.

### Conflict of interest statement

The authors declare that the research was conducted in the absence of any commercial or financial relationships that could be construed as a potential conflict of interest.

## Supplementary material

Supplementary Table 1 Primers used for real time-PCR.

Supplementary Figure 1 The photograph of RAPA nano-micelle solution.

Supplementary Figure 2 RAPA nano-micelle ophthalmic solution shows no ocular irritancy. **(A)** Ocular irritant scores for the RAPA nano-micelle ophthalmic solution at various time of the modified Draize test (New Zealand rabbits, *n* = 10/ group). **(B)** Topical reaction of cornea were observed under a slit-lamp microscope after the *in vivo* instillation of phosphate buffer saline (PBS) or the 0.1% RAPA nano-micelle ophthalmic solution for 24 h. **(C)** Histological changes in corneas at the end of the modified Draize test using Hematoxylin-Eosin (H-E) staining. Scale bar = 200 μm.

Supplementary Figure 3 RAPA nano-micelle ophthalmic solution has an anti-rejection effect advantage on conventional RAPA eyedrop. The cumulative corneal allograft survival rate was evaluated by the Kaplan-Meier method (*n* = 15–23/group). ^*^*p* < 0.05, ^***^*p* < 0.001.

Supplementary Figure 4 The transcriptional expression of CXCR2 in different MDSC populations treated by RAPA. **(A)** Transcriptional expression of CXCR2 in MDSCs isolated from RAPA nano-micelle administrated mice by real-time PCR. **(B)** The transcriptional expression of CXCR2 in M-MDSCs and G-MDSCs treated with RAPA through real-time PCR. ^**^*p* < 0.01, n.s., no significance.

Supplementary Figure 5 The expression of iNOS in different MDSC populations treated by RAPA. **(A)** iNOS protein levels in M-MDSCs and G-MDSCs was examined via western blot. **(B)** iNOS transcriptional expression in M-MDSCs and G-MDSCs was quantified by real-time PCR. ^*^*p* < 0.05, ^**^*p* < 0.01.
