# Supplementary material for: Rapamycin Nano-Micelle Ophthalmic Solution Reduces Corneal Allograft Rejection by Potentiating Myeloid-Derived Suppressor Cells' Function
Source: Front Immunol. 2018 Oct 8;9:2283. doi: 10.3389/fimmu.2018.02283 (PMC6186809; doi:10.3389/fimmu.2018.02283)
Supplement: Supplementary Table 1 — Primers used for real time-PCR. [file Table_1.DOCX]

**Supplementary Table 1. Primers used for real time-PCR**

| Gene name | Orientation | Primer sequence (5' - 3') |
| --- | --- | --- |
| GAPDH | forward | AGG GCT GCT TTT AAC TCT GGT |
|  | reverse | CCC CAC TTG ATT TTG GAG GGA |
| Arg-1 | forward | TGG GTG ACT CCC TGC ATA TCT |
|  | reverse | TTC CAT CAC CTT GCC AAT CC |
| iNOS | forward | CCG AAG CAA ACA TCA CAT TCA |
|  | reverse | GGT CTA AAG GCT CCG GGC |
| IFNγ | forward | CGG CAC AGT CAT TGA AAG CCT A |
|  | reverse | GTT GCT GAT GGC CTG ATT GTC |
| IL-17A | forward | GCT CCA GAA GGC CCT CAG ACT |
|  | reverse | CCA GCT TTC CCT CCG CAT TGA |
| IL-6 | forward | GAT GGA TGC TAC CAA ACT GGA T |
|  | reverse | CCA GGT AGC TAT GGT ACT CCA GA |
| IL-1β | forward | TGA AGT TGA CGG ACC CCA AA |
|  | reverse | TGA TGT GCT GCT GTG AGA TT |
| CXCR2 | forward | GCT CAC AAA CAG CGT CGT AG |
|  | reverse | CCA CCT TGA ATT CTC CCA TC |
| TNF-α | forward | AAT GGC CTC CCT CTC ATC AGT |
|  | reverse | GCT ACA GGC TTG TCA CTC GAA TT |
| IL-12p35 | forward | TGC CCT CCT AAA CCA CCT CAG TTT |
|  | reverse | TTT CTC TGG CCG TCT TCA CCA TGT |
