# Supplementary figures and images for: Rapamycin Nano-Micelle Ophthalmic Solution Reduces Corneal Allograft Rejection by Potentiating Myeloid-Derived Suppressor Cells' Function
Source: Front Immunol. 2018 Oct 8;9:2283. doi: 10.3389/fimmu.2018.02283 (PMC6186809; doi:10.3389/fimmu.2018.02283)

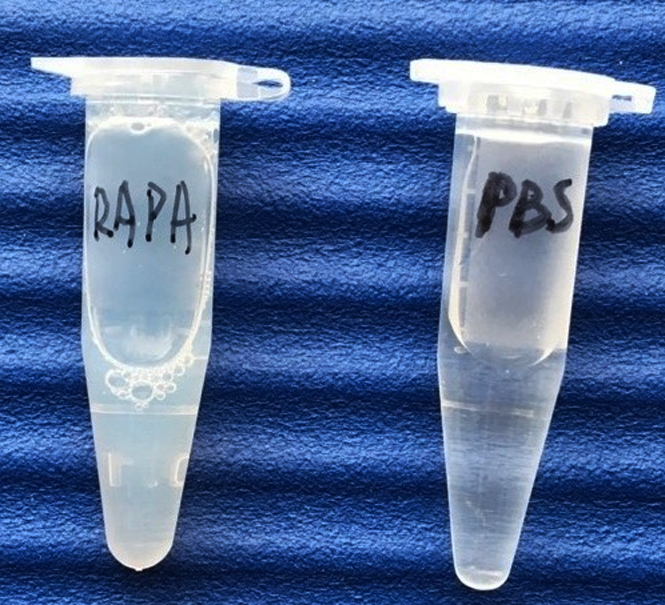

Supplement: Supplementary Figure 1 — The photograph of RAPA nano-micelle solution. [file Image_1.TIF]

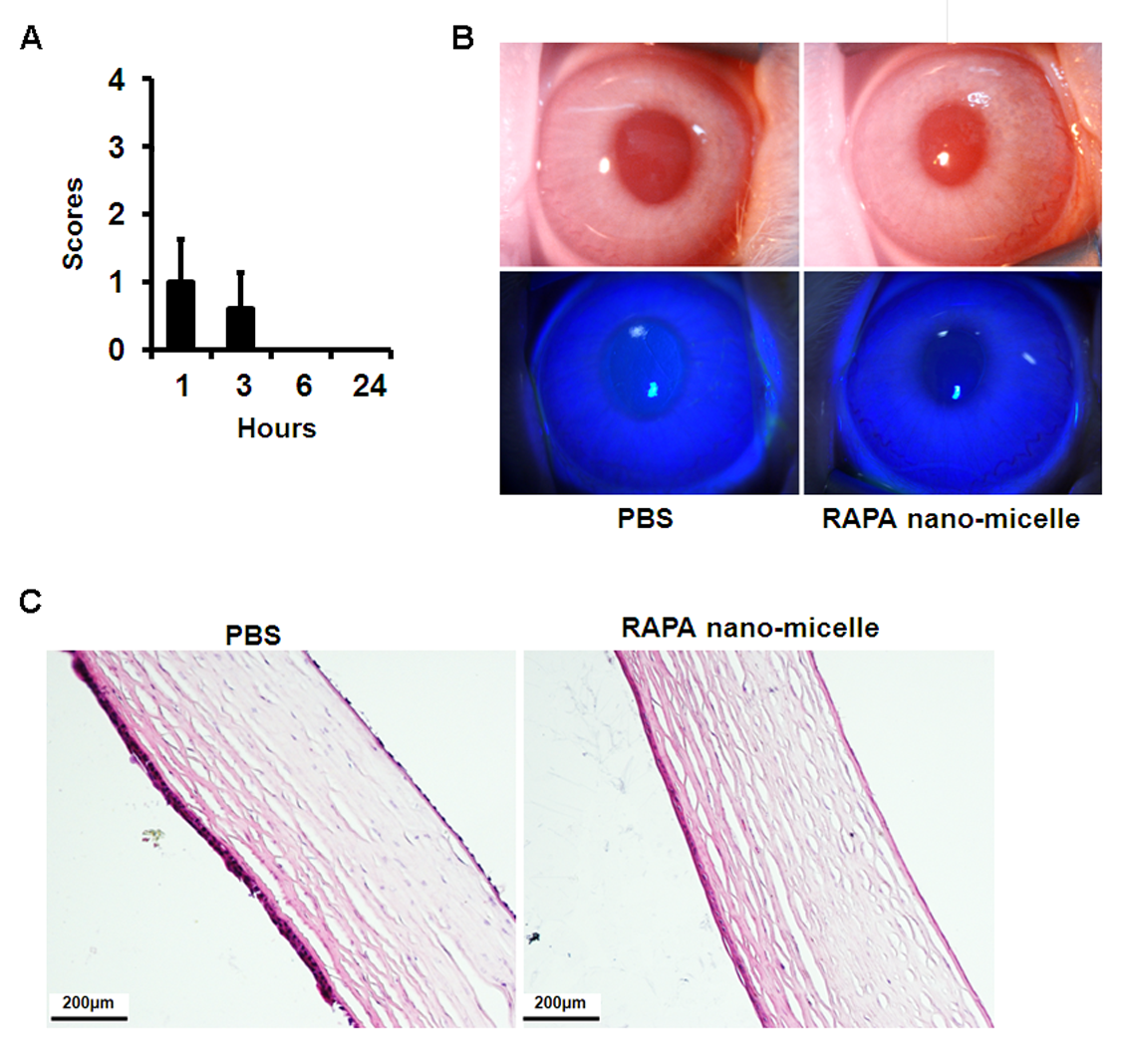

Supplement: Supplementary Figure 2 — RAPA nano-micelle ophthalmic solution shows no ocular irritancy. (A) Ocular irritant scores for the RAPA nano-micelle ophthalmic solution at various time of the modified Draize test (New Zealand rabbits, n = 10/ group). (B) Topical reaction of cornea were observed under a slit-lamp microscope after the in vivo instillation of phosphate buffer saline (PBS) or the 0.1% RAPA nano-micelle ophthalmic solution for 24 h. (C) Histological changes in corneas at the end of the modified Draize test using Hematoxylin-Eosin (H-E) staining. Scale bar = 200 μm. [file Image_2.TIF]

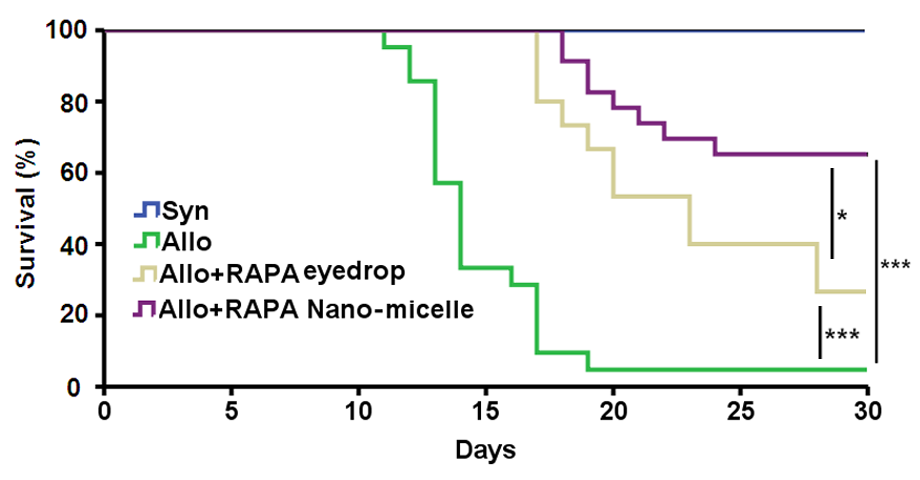

Supplement: Supplementary Figure 3 — RAPA nano-micelle ophthalmic solution has an anti-rejection effect advantage on conventional RAPA eyedrop. The cumulative corneal allograft survival rate was evaluated by the Kaplan-Meier method (n = 15–23/group). *p < 0.05, ***p < 0.001. [file Image_3.TIF]

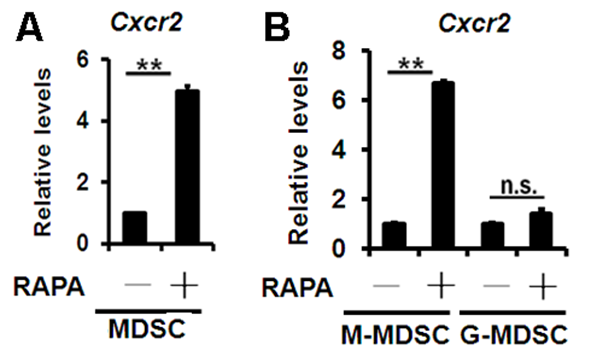

Supplement: Supplementary Figure 4 — The transcriptional expression of CXCR2 in different MDSC populations treated by RAPA. (A) Transcriptional expression of CXCR2 in MDSCs isolated from RAPA nano-micelle administrated mice by real-time PCR. (B) The transcriptional expression of CXCR2 in M-MDSCs and G-MDSCs treated with RAPA through real-time PCR. **p < 0.01, n.s., no significance. [file Image_4.TIF]

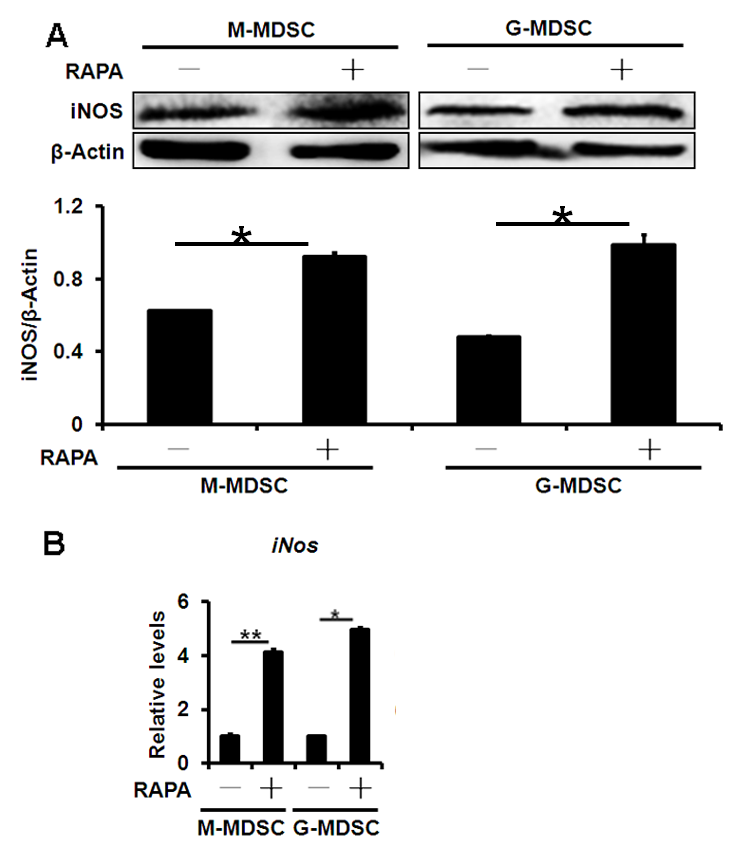

Supplement: Supplementary Figure 5 — The expression of iNOS in different MDSC populations treated by RAPA. (A) iNOS protein levels in M-MDSCs and G-MDSCs was examined via western blot. (B) iNOS transcriptional expression in M-MDSCs and G-MDSCs was quantified by real-time PCR. *p < 0.05, **p < 0.01. [file Image_5.TIF]
